# Effective microorganism water treatment method for rapid eutrophic reservoir restoration

**DOI:** 10.1007/s11356-023-31354-2

**Published:** 2023-12-08

**Authors:** Paweł Tomczyk, Paweł Stanisław Wierzchowski, Jakub Dobrzyński, Iryna Kulkova, Barbara Wróbel, Mirosław Wiatkowski, Alban Kuriqi, Witold Skorulski, Tomasz Kabat, Mirosław Prycik, Łukasz Gruss, Jarosław Drobnik

**Affiliations:** 1 Institute of Environmental Engineering, Wrocław University of Environmental and Life Sciences, Plac Grunwaldzki 24, 50-363 Wrocław, Poland; 2 Institute of Technology and Life Sciences – National Research Institute, Falenty, Poland; 3 CERIS, Instituto Superior Técnico, Universidade de Lisboa, Lisbon, Portugal; 4 Art Strefa Witold Skorulski, Wrocław, Poland; 5 DATII (Dolnośląski Akcelerator Technologii I Innowacji), Długołęka, Poland; 6 Department of Family Medicine, Wroclaw Medical University, Wrocław, Poland

**Keywords:** Effective microorganisms, Eutrophication, Biological water treatment, Water quality, Reservoir restoration

## Abstract

Since reservoirs perform many important functions, they are exposed to various types of unfavorable phenomena, e.g., eutrophication which leads to a rapid growth of algae (blooms) that degrade water quality. One of the solutions to combat phytoplankton blooms are effective microorganisms (EM). The study aims to evaluate the potential of EM in improving the water quality of the Turawa reservoir on the Mała Panew River in Poland. It is one of the first studies providing insights into the effectiveness of using EM in the bioremediation of water in a eutrophic reservoir. Samples for the study were collected in 2019–2021. The analysis showed that EM could be one of the most effective methods for cleaning water from unfavorable microorganisms (HBN22, HBN36, CBN, FCBN, FEN) — after the application of EM, a reduction in their concentration was observed (from 46.44 to 58.38% on average). The duration of their effect ranged from 17.6 to 34.1 days. The application of EM improved the trophic status of the Turawa reservoir, expressed by the Carlson index, by 7.78%. As shown in the literature review, the use of other methods of water purification (e.g., constructed wetlands, floating beds, or intermittent aeration) leads to an increase in the effectiveness and a prolongation of the duration of the EM action. The findings of the study might serve as a guide for the restoration of eutrophic reservoirs by supporting sustainable management of water resources. Nevertheless, further research should be conducted on the effectiveness of EM and their application in the remediation of eutrophic water reservoirs.

## Introduction

Water, as a resource necessary for the functioning of the environment, economy, and society (Cosgrove and Loucks 2015; Hapich et al. 2022; Wiatkowski et al. 2010), is exposed to various types of pollutants that degrade its quality (Arenas-Sánchez et al. 2016; Lin et al. 2020; Myronidis et al. 2018), such as nutrients (Lu and Tian 2017; Schoumans et al. 2014), heavy metals (Tomczyk et al. 2022; Vardhan et al. 2019), specific pollutants (Kanzari et al. 2012; Rivoira et al. 2022), and microbiological contamination (Páll et al. 2013; Paruch et al. 2019). According to the analysis of Zhang et al. (2022a), the main sources of water pollution at the global level are industrial wastewater (35.7%), rural wastewater (25.1%), municipal wastewater (18.7%), algal blooms, and agricultural activities (15.1%).

Water accumulates in various forms, but only 1% is suitable for direct human use (Oki and Kanae 2006; Khatri and Tyagi 2015). Therefore, an important issue is the purification of water from pollutants of all kinds to ensure its suitable properties for various uses (Koundouri et al. 2016; Price and Heberling 2018). Reservoirs are particularly important because they are often multifunctional and, at the same time, are a subject to various types of pollution that prevent or limit the fulfillment of their intended functions (Suwal et al. 2020; Wiatkowski et al. 2021). One of the unfavorable phenomena resulting from pollution is eutrophication caused by the influx of nutrients. It leads to a rapid growth of algae that degrade water quality (Khan and Mohammad 2014), for example, in terms of physicochemical parameters (a reduction in dissolved oxygen content) (Aguiar et al. 2011) and microbiological parameters (an increase in the number of anaerobic organisms) (Smolders et al. 2007).

Chemical, physical, and biological water treatment methods limit or prevent phytoplankton blooms in water bodies (Anawar and Chowdhury 2020; Paul et al. 2021; Lürling and Mucci 2020; Wang et al. 2019; Yin et al. 2019; Bormans et al. 2016; Jilbert et al. 2020; Bartoszek et al. 2015; Zhang et al. 2016; Ting et al. 2018).

Effective microorganisms (EM) are an example of biological (bioremediation) methods used to restore the appropriate ecological balance of the ecosystem and the ability of water to self-purify (Ateia 2016; Escudero-López et al. 2022; Wdowczyk and Szymańska-Pulikowska 2023). EM use mixtures of bacteria, actinomycetes, yeasts, and fungi with a synergistic effect to inhibit harmful bacteria by excluding competition to achieve a dominance of the effective species (Olle and Williams 2015). The term was introduced in Japan in the 1990s. EM has been studied for soil and foliar application in horticulture as an alternative to more sustainable organic agriculture that does not require fertilizers or pesticides (Talaat et al. 2015). The main advantages of using EM are their cost-effectiveness and environmental friendliness (Sharip et al. 2020). Depending on the application, EM have different forms such as loess balls (Ekpeghere et al. 2012), soil balls (Park et al. 2016), mud balls (Nugroho et al. 2017), or a liquid solution (Tommonaro et al. 2021).

Currently, EM are used not only for the treatment of eutrophic waters but also for sludge treatment, wastewater treatment, composting, medicine, livestock, forestry, and agriculture (Safwat and Matta 2021; Kour et al. 2021). Thanks to their use, it is possible, for example, to reduce sludge volume and shorten aeration time (Grabas et al. 2016; Safwat 2018), as well as to reduce odor emissions from waste and support its humification (Fan et al. 2018). Studies also found reduced nutrients and organic matter content when treating dairy wastewater (Boruszko 2019). In addition, the application of EM contributed to the improvement of vegetable production in agriculture (Olle and Williams 2015).

To date, EM have been studied in bottom sediments, in surface water of reservoirs and rivers, or in industrial waters, concerning the effects on the oxygen properties, nutrients, physical parameters, and specific pollutants of these matrices (Safwat and Matta 2021). For instance, the following phenomena were recorded after the application of EM: (i) the degradation of about 80% of microcystins in the eutrophic lakes Tsukui and Sagami in Japan (Tsuji et al. 2006); (ii) the reduction of the concentration of nitrogen and phosphorus compounds causing eutrophication in reservoirs in Hungary and India (Kamath 2007) and Poland (Jóźwiakowski et al. 2009); and (iii) the reduction of the biochemical oxygen demand (BOD), chemical oxygen demand (COD), and nitrate nitrogen (${\mathrm{NH}}_{4}^{+}$–N) in river waters (Sharip et al. 2020). On the other hand, some studies have not demonstrated significant effectiveness of the EM technology in reducing heavy metal concentrations (Ting et al. 2013), increasing the oxygenation of water bodies (Dunalska et al. 2015), or reducing the turbidity in reservoirs (Lürling et al. 2016). In this context, detailed analyses should be conducted on the extent and potential of using EM in surface water remediation projects (Mrozińska et al. 2018).

The objectives of the article are (i) an evaluation of the potential of EM to improve the water quality of the multifunctional Turawa reservoir in a separate part of the reservoir (i.e., microbiological and physicochemical parameters) located on the Mała Panew River in the Odra River basin (Southern Poland, Opole Voivodeship); (ii) an analysis of the compliance with the requirements for bathing purposes, ecological status, and trophic status of the water before and after the application of EM; and (iii) an assessment of the influence and similarity of the analyzed water parameters.

To the best of our knowledge, the study is one of a few case studies conducted in this field, providing insights into the effectiveness of using EM as a solution and Bokashi balls in the bioremediation of the multifunctional Turawa reservoir. The main challenge in the studied water body is the ongoing eutrophication process which deteriorates the reservoir water’s microbiological and physicochemical properties (Rajfur et al. 2011). To date, research on reservoir restoration has focused on the effectiveness of EM in terms of its effects on concentrations of nitrogen and phosphorus compounds. However, this manuscript focused primarily on the effects on microbiological parameters of reservoir water. On the other hand, an analysis of the relationship between the affected parameters can help develop more effective water purification strategies within specific parameter groups and develop recommendations consistent with rational water management and sustainable development goals in individual parts of large reservoirs. Pollution of reservoirs (e.g., eutrophication) is a global problem, so the issue of improving the quality of these facilities is the subject of worldwide research.

Nevertheless, in this part of Europe, there are no studies on the effectiveness of EM in improving the water quality of large reservoirs. An innovative element is the separation of a part of the reservoir used for pilot studies, which can serve different functions (e.g., recreation) for the local community. The proposed EM application method can be used as an element that supports the process of improving water quality in the reservoir.

The research on the effectiveness of EM in water treatment is a part of the project “An innovative method for improving water quality in multifunctional retention basins,” used as one of the methods for the reconstruction of the eutrophic Turawa reservoir, carried out as a part of a project awarded by the National Center for Research and Development in Poland under grant number BIOSTRATEG3/343733/15/NCBR/2018. This task was carried out in the period 01/06/2019–30/09/2021. The aim is to eliminate microbiological and chemical hazards in the water of Turawa Reservoir by using one of the innovative biological methods for lake and reservoir remediation. In connection with other tasks of the project, the following works were carried out: analyses of the microbiological parameters of the water, application of EM in the form of Bokashi balls and a liquid solution, installation of a floating dam separating part of the reservoir, and physicochemical analysis of the water.

## Materials and methods

### Field research

**Fig. 1:**
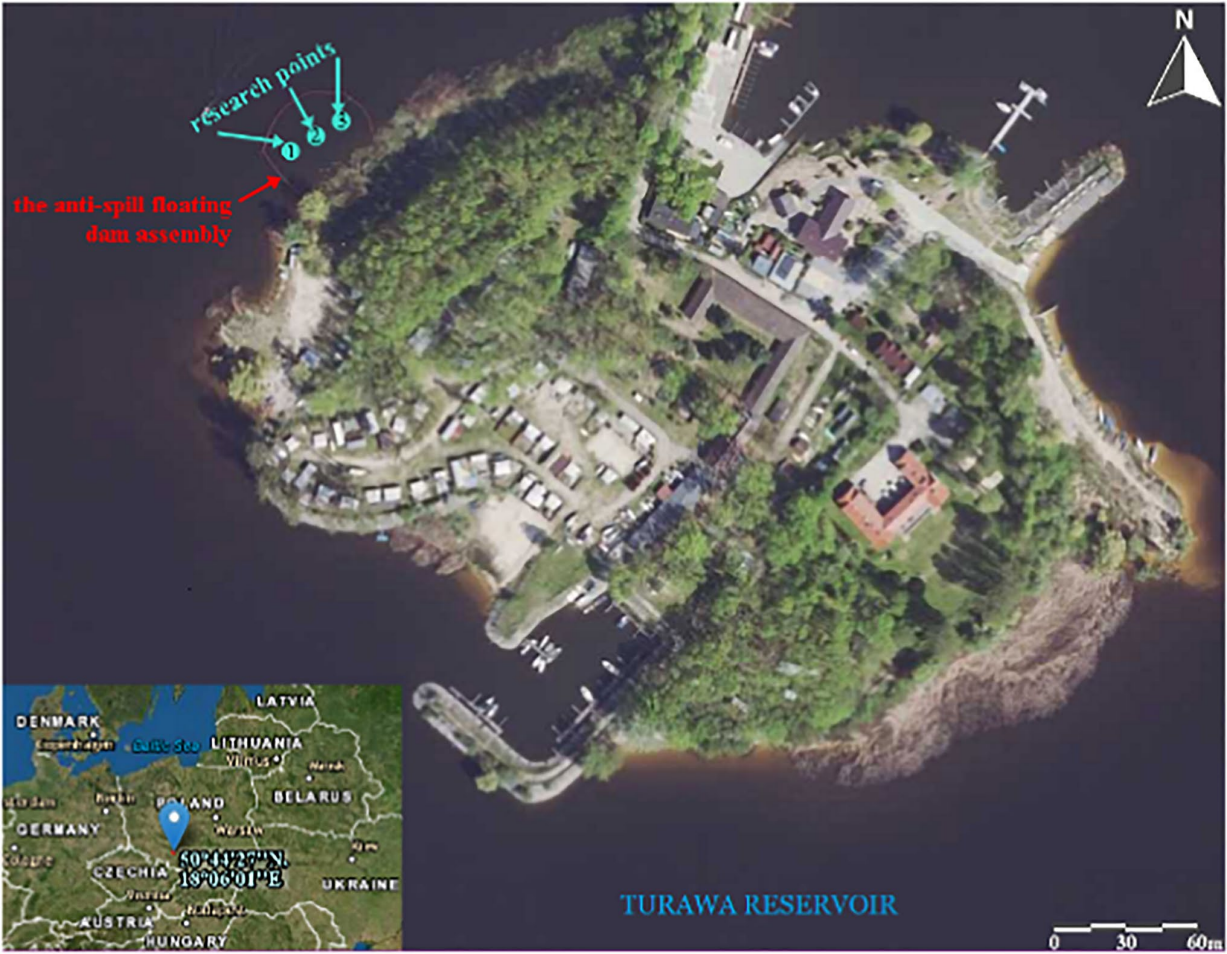
Location of research points on the Turawa reservoir (Odra river basin, southern Poland). *Source*: https://earthexplorer.usgs.gov/

The study sites were located in the Turawa reservoir with an area of 20.80 km^2^ and a maximum capacity of 107.6 million m^3^, constructed in 1933–1939 on the Mała Panew River, a right tributary of the Odra River (southern part of Poland, Opole Voivodeship) (Gruss et al. 2021; Wiatkowski and Wiatkowska 2019). Samples were collected in 2019–2021, in three replicates, from a depth of 30 and 100 cm at three study sites in the FINBOOM-type floating dam constructed in the northern part of the reservoir with an area of about 750 m^2^ (Fig. 1) (Dobrzyński et al. 2022). The location of the floating dam was determined based on recommendations of the administrator of the Turawa reservoir (area excluded from navigation), and its construction made it possible to separate a part of the reservoir (no water exchange with the rest of the reservoir) where pilot studies on the effectiveness of using EM in the surface water treatment could be conducted.

In each year of the study, microbiological and physicochemical elements were determined in three series of measurements (2 July 2019, 16 September 2019, 20 November 2019, 28 July 2020, 16 September 2020, 23 November 2020, 9 August 2021, 16 August 2021, 23 August 2021).

**Fig. 2:**
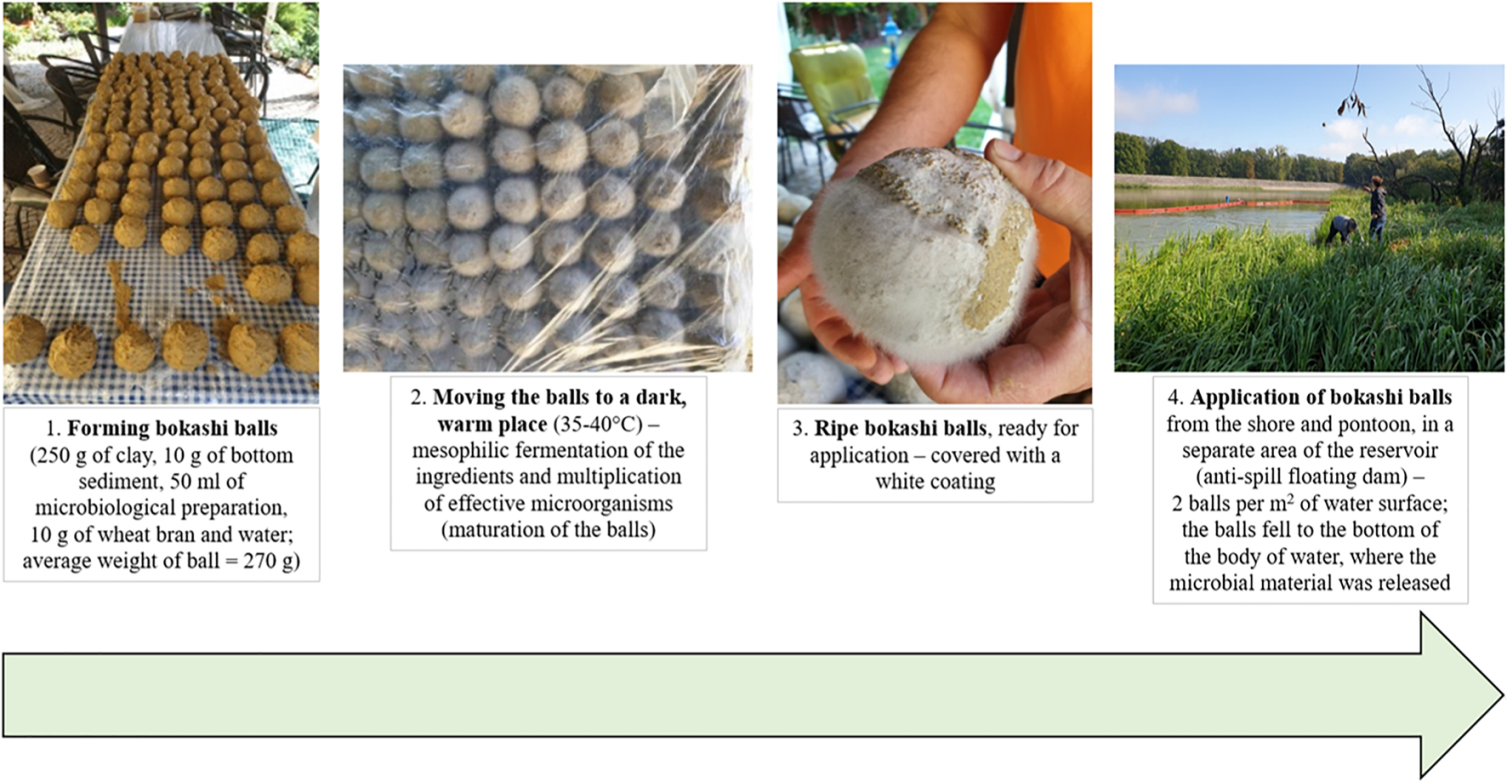
Bokashi ball application procedure in the Turawa reservoir (M. Prycik)

The application of EM took place on 9 August 2021 in two forms: Bokashi balls (Fig. 2; Table 1) under the water and in the form of liquid solution on and under the water surface. The Bokashi balls were a combination of clay, bottom sediment with a microbiological preparation SD ProBio Original and the addition of wheat bran weighing 270 g. Then, they were thrown into the separated part of Turawa reservoir at a density of 2 pieces per 1 m^2^ of water surface. As for the liquid solution, it was a stock solution of ProBio Sanit with water in a 1:10 ratio, applied with a sprayer on the water surface at a depth of 0.5 m in the amount of 10 L of the preparation per 1 m^2^ of water (area = 750 m^2^). These biopreparations contained about 80 species of bacteria — actinomycetes, yeasts, and fungi (Lactobacillus, Bifidobacterium, Pediococcus, Lactococcus, Streptococcus, Rhodopseudomonas, Aspergillus, Mucor, and Streptomyces). Their numbers in both biopreparations ranged from 10^8^ to 10^10^ colony-forming units (CFU) in 1 g (Dobrzyński et al. 2022).

**Table 1:** Taxonomic composition of the preparation with effective microorganisms, used in an application with Bokashi balls and liquid solution

Taxonomic group	Composition
Actinomycetes	*Streptomyces* and others
Aerobic fermenting fungi	*Aspergillus*, *Mucor hiemalis*, and others
Purple non-sulfur (photosynthetic) bacteria	*Rhodopseudomonas palustris*, *Rhodopseudomonas sphaerorides*
Lactic fermentation bacteria	*Bifidobacterium animalis*, *Bifidobacterium bifidum*, *Bifidobacterium longum*, *Lactobacillus acidophilus*, *Lactobacillus bulgaricus*, *Lactobacillus casei*, *Lactobacillus buchneri*, *Lactobacillus delbrueckii*, *Lactobacillus plantarum*, *Lactococcus diacetylactis*, *Lactococcus lactis*, *Streptococcus thermophilus*, *Bacillus subtilis* var. *natto*
Yeast	*Saccharomyces cerevisiae*

Other, not mentioned above; the number of individual microorganisms and the composition is a know-how secret (mother compositions of effective microorganisms developed by Matthew Wood)

### Laboratory tests

Water samples were analyzed for six microbiological parameters, including:

• The number of heterotrophic bacteria (HBN22 and HBN36) determined by the serial dilution method at 22 °C and 36 °C according to ISO 6222:1999 (ISO, 1999);
• The number of coliform bacteria (CBN) and fecal coliform bacteria (FCBN) determined using the multiple tube fermentation technique and calculated as the most probable number in 100 mL of the water tested (Bachtiar 2002);
• Fecal enterococci count (FEN) determined by membrane filtration method according to ISO 7899–1 (ISO 2000);
• *Salmonella* spp. in 1 L of water determined using the membrane filtration method described in ISO 19250:2010 (ISO 2010).

The study also analyzed selected physicochemical parameters of water, including pH (potentiometric method), electrolytic conductivity (EC; conductometric method), total nitrogen (*TN*; spectrophotometric method), nitrate nitrogen (NO_3_–N; spectrophotometric method), phosphate phosphorus (PO_4_–P; spectrophotometric method), total phosphorus (*TP*; spectrophotometric method), dissolved oxygen (DO; electrochemical sensor), 5-day biochemical oxygen demand (BOD_5_; dilution method), and chemical oxygen demand (COD; permanganate method). Each water determination was repeated three times (Szymańska-Pulikowska et al. 2023; Puchlik et al. 2022; MIP 2021; MHP 2019).

### Data analysis

**Fig. 3:**
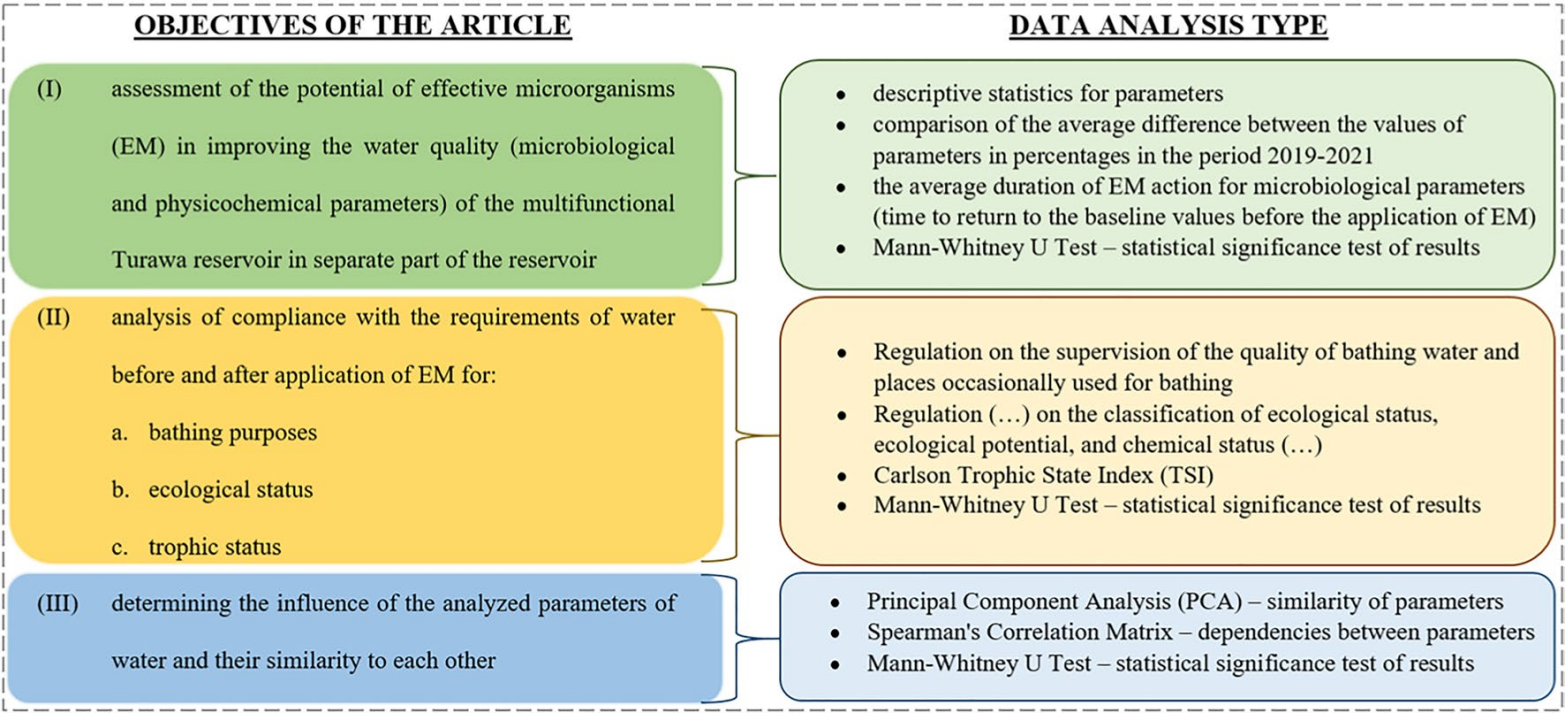
Associating article objectives with types of data analysis

The article includes the following analysis of the results (statistical significance for *p* < 0.05) (Tomczyk et al. 2022; Wiatkowski et al. 2021; Tomczyk and Wiatkowski 2021; MIP 2021; MHP 2019; Ji et al. 2020):

• Descriptive statistics for microbiological and physicochemical parameters — a comparison of the results before and after application of EM (minimum, maximum, standard deviation, median, mean) — Objective I (see Fig. 3), “Descriptive statistics and statistical significance test of results” section;
• Mann–Whitney *U* test — for two groups of measurement series, i.e., before and after the application of EM (for the entire research period 2019–2021, as well as a comparison of the results for the series from 2021 — the first series immediately before the application, the second — 1 and 2 weeks after the application, i.e., on 9, 16, and 23 August); the test compares the medians for the selected groups and the data have a non-linear distribution — Objectives I, II, and III, “Descriptive statistics and statistical significance test of results,” “Comparison of the effectiveness of effective microorganisms before and after their application,” and “Correlations between microbiological and physicochemical parameters” sections;
• An evaluation of the effectiveness of EM on microbiological and physicochemical parameter values (a comparison of the average difference between parameter values in percent over the period 2019–2021, the average duration of the effect of EM for microbiological parameters — August 2021 — time to return to baseline values before application of EM as of 9 August 2021, assuming it is proportional to the rate of change between parameter values when comparing August 2021 series before and after application of EM) — Objective I, “Influence on microbiological parameters immediately before and after application” section;
• An assessment of compliance with microbiological requirements for bathing waters — based on the Regulation on monitoring the quality of bathing waters and places occasionally used for bathing (MHP 2019); microbiological parameters (CBN and FEN) are assessed on a 2-point scale, i.e., compliance — 1 point, non-compliance — 0 point; final rating is expressed on a scale from 0 to 1 and indicates the percentage of samples for which bathing water quality requirements were met (average 0.00–0.50 point — non-compliance, 0.51–1.00 — compliance); survey series for the period 2019–2021 and three series from 2021 were compared — Objective IIa, “Ecological status of physicochemical parameters and meeting the microbiological requirements of water for bathing purposes” section;
• A determination of the ecological status of physicochemical parameters — following the Regulation of the Minister of Infrastructure of 25 June 2021 on the classification of ecological status, ecological potential, and chemical status and on the method of classifying the status of surface water bodies and on environmental quality standards for priority substances (MIP 2021); the physicochemical elements are assessed on a 3-point scale, depending on the abiotic type of the water body (in this case, type 0, i.e., the stream of undetermined type — reservoir), and for each of the results from 2019–2021 they were weighted accordingly, i.e., Class I (very good physical–chemical condition) — 1 point, Class II (good condition) — 2 points, Class III (less than good condition) — 3 points, and then the average was calculated for the results from the entire study period; the final classification of the results is as follows: 1.00–1.66 points — 1st class, 1.67–2.33 points — class II, 2.34–3.00 points — 3rd class — Objective IIb, “Ecological status of physicochemical parameters and meeting the microbiological requirements of water for bathing purposes” section;
• A determination of trophic status (Wiatkowski et al. 2021; Ji et al. 2020) before and after the application of EM by the Carlson trophic state index (*TSI*) calculated from the average concentrations of *TN* and *TP* according to Formulas 1–3 — Objective IIc, “Ecological status of physicochemical parameters and meeting the microbiological requirements of water for bathing purposes” section:
  1 $$TSI\left(TP\right)=10 \bullet \left(9.40+1.62 \mathrm{In}\left(TP\right)\right)$$
  2 $$TSI\left(TN\right)=10 \bullet \left(5.24+1.86 \mathrm{In}\left(TN\right)\right)$$
  3 $$TSI = \frac{TSI \left(TP\right)+TSI (TN)}{2}$$
• A principal component analysis (PCA) for all parameters analyzed; additionally, Kaiser–Meyer–Olkin measure of sampling adequacy, Bartlett’s test of sphericity, communalities, explained total variance, Scree plot, and component matrix-destination — Objective III, “Correlations between microbiological and physicochemical parameters” section;
• Spearman’s correlation matrix — a comparison of the direction and strength of correlation between each parameter for a non-linear distribution — Objective III, “Correlations between microbiological and physicochemical parameters” section.

The data obtained were analyzed using the following software: IBM SPSS Statistics 26, Statistica 13, Origin 2021b, and Excel 2013.

Figure 3 compares the formulated goals of the article with the data analyses described above.

## Results

### Descriptive statistics and statistical significance test of results

Data analysis for the period before EM application (2019, 2020, and 9 August 2021) and after EM application (16 and 23 August 2021) is presented in Table 2. It shows that the long-term average outcome difference between the periods ranged from − 72.04% (HBN22) to 137.04% (FEN). Statistical significance of the results, performed with the Mann–Whitney *U* test, was recorded for 9 of 14 parameters, i.e., HBN22 (− 72.04%), HBN36 (− 71.20%), NO_3_–N (− 57.19%), PO_4_–P (− 49.43%), COD (− 12.23%), pH (5.67%), DO (20.29%), FCBN (33.33%), CBN (34.25%), and FEN (137.04%). Throughout the study period, no Salmonella bacteria were found in the water and the parameter did not change over time, so it was not included in further analyses.

**Table 2:** Descriptive statistics for microbiological and physicochemical parameters before and after EM application in the Turawa reservoir

Statistics	Period	Parameter (unit)													
		HBN22 (CFU∙mL^−1^)	HBN36 (CFU∙mL^−1^)	CBN (CFU∙mL^−1^)	FCBN (CFU∙mL^−1^)	FEN (CFU∙mL^−1^)	pH (-)	EC (µS∙cm^−1^)	*TN* (mg∙L^−1^)	NO_3_–N (mg∙L^−1^)	PO_4_–P (mg∙L^−1^)	*TP* (mg∙L^−1^)	DO (mg∙L^−1^)	BOD_5_ (mg∙L^−1^)	COD (mg∙L^−1^)
Min	Before EM	180	50	23	15	3	6.70	202	1.31	0.060	0.000	0.03	0.00	0.70	1.20
	After EM	2100	1500	75	75	9	6.88	203	1.48	0.110	0.009	0.09	7.30	1.70	6.70
Max	Before EM	985,000	755,000	2420	2420	460	9.90	491	12.30	4.210	0.390	11.50	13.10	54.00	320.10
	After EM	110,000	71,000	2420	2420	460	10.30	507	12.09	1.710	0.100	1.07	12.30	9.80	30.80
Std. deviation	Before EM	192,694	181,379	920	893	86	0.65	39	2.04	0.824	0.052	1.52	2.10	6.32	38.81
	After EM	24,667	18,005	723	709	126	0.92	42	2.29	0.437	0.017	0.25	1.33	2.20	6.55
Mean	Before EM	79,013	77,696	760	677	54	8.51	403	3.78	1.140	0.075	0.65	8.30	5.27	17.47
	After EM	22,093	22,374	1020	902	128	8.99	419	3.89	0.488	0.038	0.39	9.98	4.89	15.34
Mean difference after and before EM		* − 72.04%*	* − 71.20%*	*34.25%*	*33.33%*	*137.04%*	*5.67%*	3.88%	2.84%	* − 57.19%*	* − 49.4%*	* − *40.58%	*20.29%*	* − *7.11%	* − 12.23%*
Mann Whitney *U* Test	*U*-value	1227	1113	1375	1321.5	1212	787	870	1039.5	1897.5	1764	1356.5	537.5	961.5	755.5
	*z*-score	− 4.19585	− 4.65108	− 3.59558	− 3.81111	− 4.25225	− 2.8597	− 1.65712	0.07906	3.96203	3.81245	0.65932	− 4.3344	− 0.9678	− 2.42999
	*p*	< 0.00001	< 0.00001	0.00032	0.00014	< 0.00001	0.00424	0.09692	0.93624	0.00008	0.00014	0.50926	< 0.00001	0.33204	0.0151

Designations in the table: italics — values statistically significant for *p* ≤ 0.05

### Comparison of the effectiveness of effective microorganisms before and after their application

#### Influence on microbiological parameters immediately before and after application

The comparison of the results immediately before and after the application of EM (before application — 9 August 2021, after application — 16 and 23 August) is summarized in Table 3. All are statistically significant according to the Mann–Whitney *U* test. In most cases, there was a decrease in parameter values (exceptions: CBN in point 1 at a depth of 30 cm; HBN36, FCBN, and FEN in point 1 at a depth of 100 cm; and HBN22 and HBN36 in point 2 at a depth of 100 cm — an increase from 17.96 to 99.16%). This reduction ranged from 19.39% (FCBN, point 1 at 30-cm depth) to 88.97% (CBN, point 3 at 100-cm depth). The overall reduction of parameters for all points was decreasing: 58.38% — HBN22, 55.77% — FEN, 48.88% — HBN36, 47.22% — FCBN, and 46.44% — CBN. This is also reflected in the average duration of the action of EM, which ranges from 423 to 819 h (FEN and CBN, respectively). The results obtained demonstrate the effectiveness of EM in most cases, lasting up to more than 1 month after application, with an average reduction effect of at least 45%.

**Table 3:** The average percentage effectiveness of EM activity and the average time of EM activity in reducing the value of microbiological parameters in the analyzed water test points of the Turawa reservoir

Parameters	Description	Microbiological parameters				
		HBN22	HBN36	CBN	FCBN	FEN
Average percentage effectiveness of EM at test points	1 (30 cm)	− 60.55%	− 38.21%	40.00%	− 19.39%	− 37.91%
	1 (100 cm)	− 73.13%	17.96%	0.00%	28.57%	89.96%
	2 ( 30 cm)	− 47.50%	− 92.13%	− 72.18%	− 65.99%	− 86.38%
	2 (100 cm)	99.16%	62.45%	− 76.77%	− 72.18%	− 79.23%
	3 (30 cm)	− 63.76%	− 67.73%	− 13.89%	− 79.59%	− 37.74%
	3 (100 cm)	− 71.22%	− 60.71%	− 88.97%	− 73.16%	− 80.66%
	Overall^1^	− 58.38%	− 48.88%	− 46.44%	− 47.22%	− 55.77%
Average duration of action of EM in total in reducing parameter values^2^	Days	19.52	19.67	34.13	30.47	17.64
	Hours	468.5	472.1	819.2	731.3	423.4
	Minutes	28,110	28,323	49,152	43,878	25,401
Mann–Whitney *U* test	U-value	*65.5*	*84.0*	*77.5*	*74.5*	*55.0*
	*z*-score	*3.03731*	*2.35199*	*2.65764*	*2.75256*	*3.36951*
	P	0.00236	0.01428	0.00782	0.00596	0.00076

Italics — statistically significant values for *p* ≤ 0.05

^1^Mean value for individual parameters from the series immediately before and after the application of EM

#### Ecological status of physicochemical parameters and meeting the microbiological requirements of water for bathing purposes

Table 4 presents the standardized results of the ecological status of physicochemical parameters concerning meeting the microbiological requirements of water for bathing purposes and the trophic status before and after the application of EM.

**Table 4:**
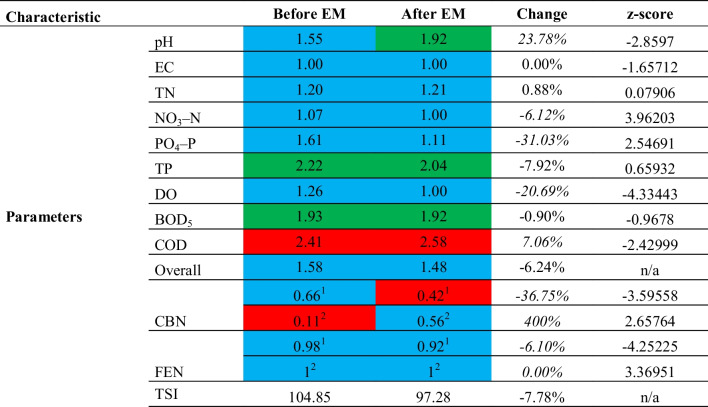
Summary of the ecological status results for physicochemical parameters, microbiological requirements compliance, and trophic status of Turawa reservoir water before and after application of EM

Designations in the table: blue color — class I of physicochemical condition (very good condition) or compliance with microbiological requirements, green color — class II of physicochemical condition, and red color — less than good condition or non-compliance with microbiological requirements

Italic — statistically significant values for *p* ≤ 0.05, *n/a* — not applicable

^1^Values for the whole series of measurements (2019–2021)

^2^Values for the series immediately before and after EM application

As for the physicochemical parameters for the whole study period 2019–2021, the statistical significance of the results for pH, NO_3_–N, PO_4_–P, DO, and COD was found. Among the mentioned parameters, after the use of EM, the ecological status improved for NO_3_–N (by 6.12%), PO_4_–P (by 31.03%), and DO (by 20.69%), and worsened for pH (by 23.78%) and COD (by 7.06%). Within the classification of average ecological condition, the class change was observed only in the case of pH (from class I to II, i.e., from very good to good condition). In contrast, in the other cases, the results had the same class both before and after the application of EM. The factor that worsened the results was the COD — physicochemical condition below good. Good status was found for *TP* and BOD_5_, and pH — very good status before EM application and good status after application (the objective of the Water Framework Directive is to achieve at least good status in surface waters) until the end of a certain planning period, so these results have no impact. The remaining elements assessed are within the allowable limits for 1st-class ecological status. An overall comparison of the physicochemical results shows that the ecological status improved by only 6.24% after using EM.

For the evaluated microbiological parameters (CBN and FEN), the statistical significance of the results was established, taking into account both the compliance with the requirements for bathing purposes in the series immediately before and after the application of EM (August 2021) and in the series for the entire analyzed research period (2019–2021). It is worth noting that the results for CBN in the series immediately before and after the application of EM improved by 400% in terms of requirements (compliance in 11% before application and 56% after application); in the case of FEN, there were no differences between the results (100% compliance). Considering the whole series of measurements, the results for CBN deteriorated by 36.75% (fulfillment of requirements in 66% of samples before application and 42% after application), and for FEN — by 6.10% (98% before application and 92% after application).

The general trophic condition improved after the treatment with EM — the Carlson index (*TSI*) decreased by 7.78%. Due to the high concentrations of *TP* and *TN*, the waters of Turawa reservoir are eutrophic or hypereutrophic, depending on the classification (Wiatkowski et al. 2021). The *TSI* values before and after the application of EM equaled: for *TN* — 115.37 and 116.00 (eutrophic or hypereutrophic) and *TP* — 94.34 and 78.55 (eutrophic).

### Correlations between microbiological and physicochemical parameters

The analysis of the communalities shows that the values for the analyzed parameters after extraction using principal component analysis range from 0.610 (BOD_5_) to 0.949 (NO_3_–N). For BOD_5_, FEN (0.633), pH (0.674), and *TP* (0.728) values are below 0.800. All parameters were included in the principal component analysis.

The analysis revealed four components with eigenvalues greater than 1. The first principal component (PC1) explained 36.01% of the variance, the second (PC2) — 18.74%, the third (PC3) — 17.88%, and the fourth (PC4) — 9.16% (81.79% of the cases).

**Fig. 4:**
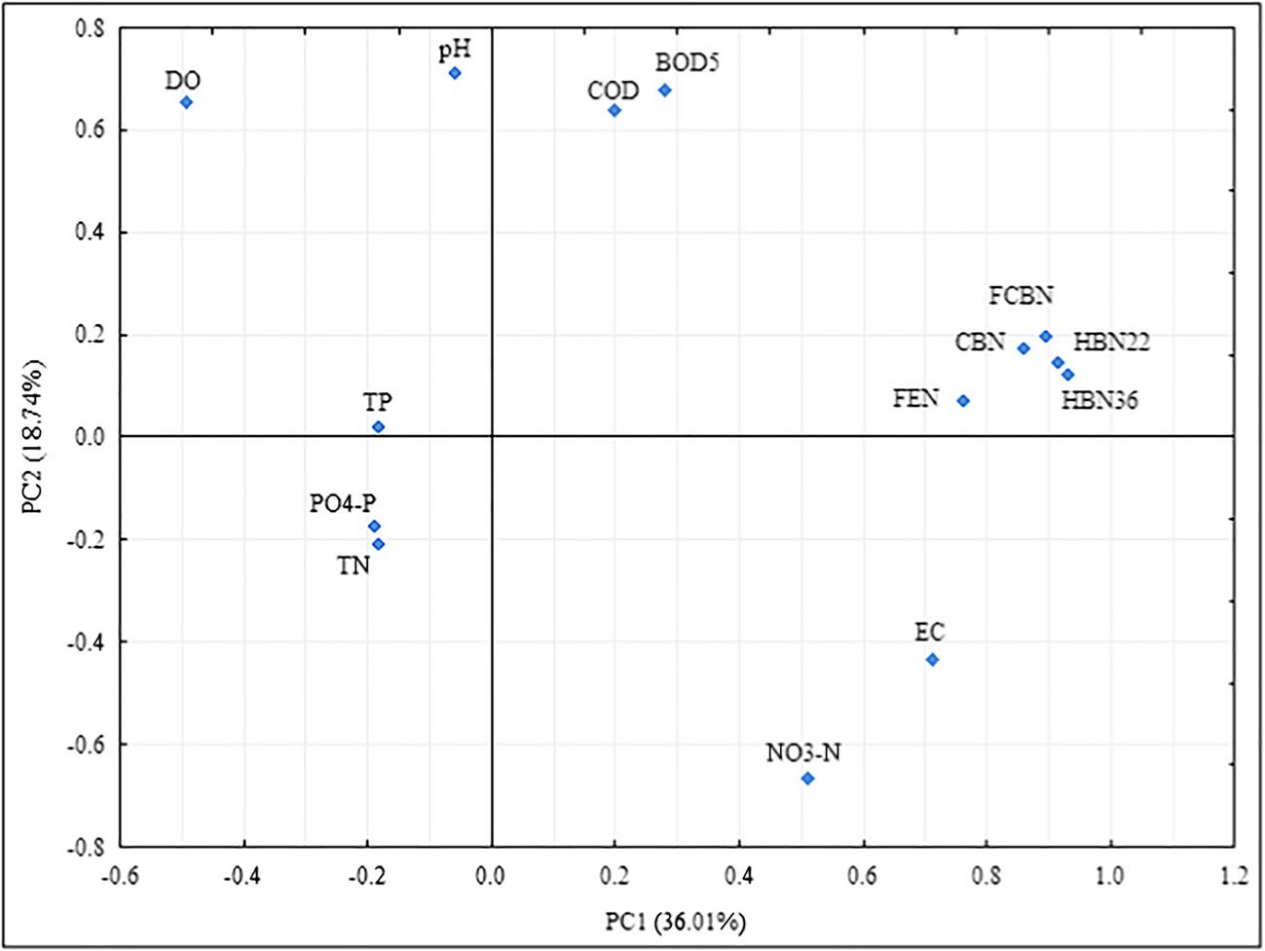
Principal component analysis for the analyzed physicochemical and microbiological parameters of the Turawa reservoir in 2019–2021

The component matrix shows that 6 parameters are most strongly correlated with the PC1 — HBN22, HBN36, CBN, FCBN, FEN, and EC (0.910. 0.928, 0.857, 0.891, 0.759, and 0.717); with the PC2 — 5 parameters: pH, NO_3_–N, DO, BOD_5_, and COD (0.644, 0.713, 0.576, 0.666, and 0.710); with the PC3 — 2 parameters: PO_4_–P and *TP* (0.931 and 0.819); and with the PC4 — 1 parameter: *TN* (0.848). These results are shown for the first and second components in Fig. 4.

The results for microbiological parameters are most similar (FEN, CBN, FCBN, HBN22, and HBN36; the maximum difference in the distance between the points on the axes: *X* = 0.169, *Y* = 0.127) for selected nutrients (*TP*, PO_4_–P, and *TN*; difference *X* = 0.006, *Y* = 0.228) and some oxygen parameters (COD and BOD_5_; difference *X* = 0.080, *Y* = 0.039). pH, DO, EC, and NO_3_–N differ from the other results for the first and second components.

**Fig. 5:**
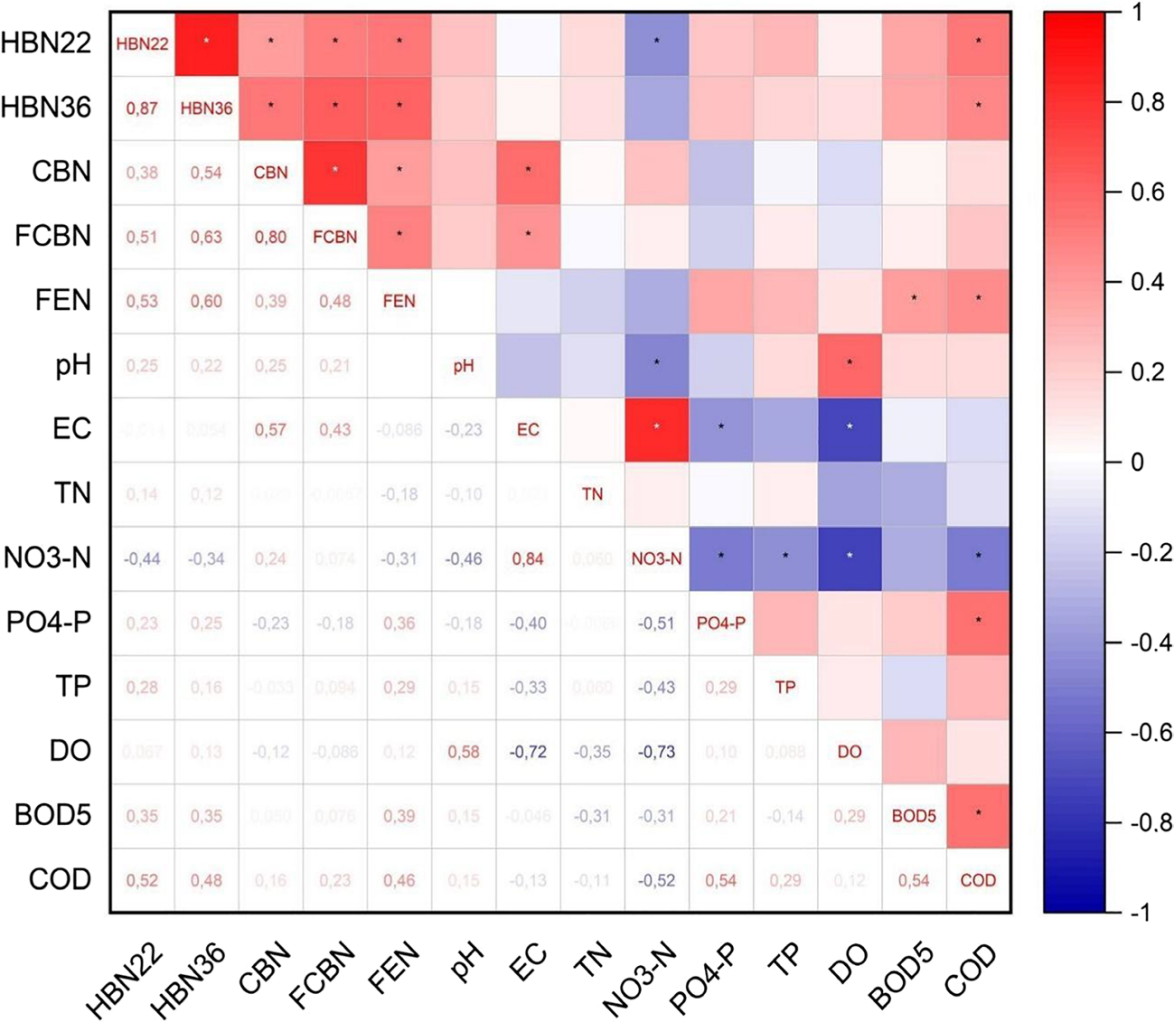
Spearman correlation matrix for microbiological and physicochemical water parameters from Turawa reservoir (asterisk — results significant for *p* ≤ 0.05)

Spearman correlation matrix (Fig. 5) was performed to investigate possible relationships between parameters. Statistical significance was found between all microbiological parameters (HBN22, HBN36, CBN, FCBN, FEN) and also between EC/CBN, EC/FCBN, NO_3_–N/HBN22, NO_3_–N/pH, NO_3_–N/EC, PO_4_–P/EC, PO_4_–P/NO_3_–N, *TP*/NO_3_–N, DO/pH, DO/EC, DO/NO_3_–N, BOD_5_/FEN, COD/HBN22, COD/HBN36, COD/FEN, COD/NO_3_–N, COD/PO_4_–P, and COD/BOD_5_.

Assuming that *R* ≥  ± 0.70 means a strong correlation; it occurred in the following cases: HBN36/HBN22 (0.87), FCBN/CBN (0.80), NO_3_–N/EC (0.84), DO/EC (− 0.72), and DO/NO_3_–N (− 0.73). It means that there was a strong statistically significant correlation for only five pairs of parameters — directly proportional for positive values and inversely proportional for negative values.

## Discussion

To date, few microbiological and physicochemical analyses of surface water have been conducted using EM for river or reservoir remediation (Park et al. 2016; Li et al. 2020; Zhao et al. 2013; Sitarek et al. 2017; Dondajewska et al. 2019). As shown in the summarized results in Table 5, when comparing the results before and after the application of EM, the similarity between the results from this study and literature was observed in 6 out of 9 microbiological and physicochemical parameters in terms of changes in the values of specific parameters (CBN, FEN, *TP*, PO_4_–P, NO_3_–N, pH). The highest number of results in all studies was recorded for *TP* — conducted in China in artificial wetlands in Kunshan in Jiangsu Province (Li et al. 2020) and artificial lake in Guilin City in Guangxi Province (Zhao et al. 2013)), in Poland in Muchawka reservoir in Siedlce City (Sitarek et al. 2017) and Konin lake near Konin City (Dondajewska et al. 2019), and in South Korea in Dalseong wetland in Daegu City (Park et al. 2016) — the reduction after using EM ranged from 13.75 to 86.87% (in this study: 45.08%). For *TN*, slightly fewer studies were performed, and the changes in the values of this parameter after using EM ranged from − 57.36 to 2.84% (decrease in 4 studies, increase in 1). The decrease in the parameter values after the application of EM was visible in the case of CBN, FEN, NO_3_–N, and PO_4_–P (respectively, by 58.83%, 67.89%, 22.47%, and 37.70%). In the case of pH, there was a 1.31% increase in the average value. Divergent results between the cited test results and those of other conducted studies were found for EC and DO (in each case, a decrease in values in the other study and an increase in this one — with the direction of change being negative for EC in the studies conducted, and positive for DO; the differences between the values before and after the application of EM in this study were at the margin of error of the measurement methods, i.e., up to 5%, so they are most likely not significant).

**Table 5:** Comparison of the effectiveness of EM activity concerning selected microbiological and physicochemical parameters of water, based on a literature review

Parameter (unit)	Before EM	After EM	% difference	References
CBN (CFU·mL^−1^)	290	83.5	− 71.21%	Sitarek et al. (2017)
	1677^1^	898^1^	− 46.45%	This study
FEN (CFU·mL^−1^)	462.5	91	− 80.32%	Sitarek et al. (2017)
	119^1^	53^1^	− 55.46%	This study
pH (-)	8.37	8.67	3.59%	Sitarek et al. (2017)
	7.23^1^	7.30^1^	0.98%	This study
EC (μS/cm)	388	311.5	− 19.72%	Dondajewska et al. (2019)
	207^1^	209^1^	0.97%	This study
NO_3_–N (mg·L^−1^)	-	-	− 34.50%	Li et al. (2020)
	0.515^1^	0.461^1^	− 10.43%	This study
*TN* (mg·L^−1^)	3.68	3.75	1.81%	Dondajewska et al. (2019)
	4.50	3.30	− 26.67%	Park et al. (2016)
	-	-	− 21.37%	Li et al. (2020)
	-	-	− 57.36%	Zhao et al. (2013)
	3.84^1^	3.42^1^	− 10.94%	This study
PO_4_–P (mg·L^−1^)	0.11	0.055	− 50.00%	Dondajewska et al. (2019)
	0.063^1^	0.047^1^	− 25.40%	This study
*TP* (mg·L^−1^)	0.19	0.16	− 17.24%	Dondajewska et al. (2019)
	0.27	0.17	− 38.27%	Sitarek et al. (2017)
	0.55	0.23	− 58.18%	Park et al. (2016)
	-	-	− 13.74%	Li et al. (2020)
	-	-	− 86.87%	Zhao et al. (2013)
	0.25^1^	0.14^1^	− 45.08%	This study
DO (mg·L^−1^)	9.8	9.6	− 2.04%	Dondajewska et al. (2019)
	7.89^1^	8.29^1^	5.07%	This study

^1^Value for the series directly before and after EM application

Regarding the duration of the action of EM, in the studies conducted at Kunshan Irrigation and Drainage Experimental Station (Li et al. 2020), a reduction of pollutants in the treated water was observed for each of the parameters considered below (NO_3_–N, *TN*, *TP*), throughout the study period (8 days) after the application of EM. However, in a liquid form (80% water, 10% nutrient solution, and 10% basic EM), the greatest and most permanent reduction of nutrients was achieved in combination with constructed wetlands. In this case, 8 days after the application of these solutions, there was even more than 92% of reduction (for comparison for NO_3_–N: EM application — reduction by a maximum of 66%, constructed wetlands — by 83%).

In Chinese studies conducted in an artificial lake (polluted by municipal wastewater) in Guilin City, Guangxi Province (Zhao et al. 2013), a pollution reduction was observed after the application of a comprehensive treatment, i.e., a combination of the application of EM in liquid form (concentration 0.2%), the use of phytoplankton-feeding fish, floating islands with macrophytes, and intermittent aeration of the reservoir. A reduction of *TN* and *TP* (by 54.18–60.54% and 85.76–87.98%, respectively) was observed during most of the study period (i.e., from 25 February 2012 to 2 June 2012). The only factor that disrupted the process was rainfall with a certain pollutant load.

In a study conducted in the constructed wetland in Kunshan, Jiang Province, China (Li et al. 2020), a decrease in the concentrations of *TN* and *TP* was observed throughout the measurement period (30 days) — water with EM-enriched soil balls (Bokashi balls) with 0.75% hardener was used.

In analyses conducted at Lake Konin in Poland (Dondajewska et al. 2019), after the application of EM (in the form of mud balls) in 2014, a gradual decrease of *TP* was observed compared to the values of 2011–2013 (by about 17.2%), while the median concentration of the parameter remained unchanged in 2015. For *TN*, the median value was the lowest in 2014 (by 10.4% on average, compared to 2011–2013), but higher concentrations of the parameter were recorded in 2015 (27.3% compared to 2014). It should be noted that the greatest changes observed after the deployment of EM were in species composition and phytoplankton abundance, which underwent a positive change in 2014. Such an effect lasted from April 2014 to June 2015 and, from July 2015, a return to the previous taxonomic structure characteristic of eutrophic waters was observed (however, the number of these taxa was lower than before the reconstruction of the lake).

A decrease in the content of microbiological parameters in the tests at the Mukhavka reservoir in Poland (Dondajewska et al. 2019) was observed only in the first series of measurements after the application of EM in liquid form (3 June 2013). In subsequent series of tests (from 9 July 2013), the content of both CBN and FEN fluctuated, indicating a short-term effect of the application of EM on microbiological parameters.

As shown by the above and other studies on using EM as a biological method to improve surface water quality, their main activity is related to their role in aquatic ecosystems. Since most of these organisms are decomposers that break down organic matter contained in bottom sediments, the direct effect of their use in water reservoirs or rivers is a reduction in the thickness of bottom sediments (Mazur 2020), as well as a reduction in the levels of various forms of nitrogen and phosphorus in the water column. It results from the conversion of the organic compounds used to inorganic compounds and the consumption of oxygen needed to carry out this process (Park et al. 2016). According to research, an additional, further effect of EM is also a change in the species composition of the flora of water reservoirs, especially an increase in the richness of phytoplankton, which is a positive phenomenon (Mazurkiewicz et al. 2020). Some studies have confirmed that immobilized EM can effectively remove microcystins (cyanobacterial toxins) up to 2 months after their application (Tsuji et al. 2006; Zhang et al. 2022b). However, as research points out, EM alone cannot improve water quality. They do not produce the desired long-term effects in terms of reducing nutrients and increasing the transparency and oxygen content of water (Dondajewska et al. 2019; Li et al. 2020). They are recommended as supporting methods for other biological, physical, or chemical methods of surface water restoration (e.g., phytoremediation, use of synthetic zeolites, or injection of salt into bottom sediments) (Gao et al. 2023; Tarczyńska et al. 2001). A particularly recommended biological method for controlling water blooms in reservoirs is the creation of ecotone zones consisting of macrophytes that prevent the resuspension of sediments (containing, among other things, nitrogen and phosphorus compounds that are food for algae that cause eutrophication) that accumulate harmful substances in their tissues (e.g., heavy metals) and that perform important ecological functions (e.g., as shelter for other groups of organisms) (Chen et al. 2019; O’Hare et al. 2018; Wiatkowski 2015).

The results presented in this article demonstrate the described process of decomposition of organic matter by EM, which leads to lower levels of PO_4_–P, *TP*, NO_3_–N, and *TN* immediately after application. In addition, the microbiological structure was rebuilt, as evidenced by the reduction in the number of analyzed bacteria in most of the collected water samples, which, among others, pose a risk to human health after consumption (e.g., diseases of the digestive and urinary systems) (Braiek and Smaoui 2019; Zhi et al. 2020).

The previously presented strong correlation between HBN36 and HBN22 and FCBN and CBN is related to the identical method of determination between the pairs of parameters, which considered the same groups of bacteria but cultured at different temperatures (HBN36 and HBN22) or similarly (CBN — all coliforms, FCBN — fecal coliforms only) (ISO 1999; Bachtiar 2002). As for the correlation between EC and NO_3_–N and EC and DO, a higher EC value indicates more saturation of water with ions and often more water pollution. For this reason, the correlation is directly proportional for the first pair and inversely proportional for the second (Zhang et al. 2022c). The correlation between DO and NO_3_–N results from the decomposition of organic matter, which is mainly carried out by algae as a part of the eutrophication process — when water highly enriched in NO_3_–N and DO is consumed (especially in the summer months when temperature is high and light is abundant). Thus, there is an inverse correlation (Lv et al. 2022; Sun et al. 2022). It is worth noting that, as shown in other studies on the eutrophic Turawa reservoir (Czerniawska-Kusza and Kusza 2011; Buta et al. 2023), nitrogen compounds in the water come mainly from external sources of agricultural origin, while phosphorus compounds come from internal sources, i.e., resuspension of sediments.

Moreover, according to research results for the period 1998–2020 (Buta et al. 2023), the trophic status of the Turawa reservoir, expressed by the Carlson index (Carlson 1977; Carlson & Simpson 1996) and the Vollenweider and Kajak criterion (Vollenweider 1965; Kajak 2001) is classified as eutrophic, and according to the Trophic Level Index (Abell et al. 2020; Ding et al. 2021) — as supertrophic. It is worth adding that *TN* was on average 10 times more responsible for eutrophication than *TP*, taking into account the values of dangerous loads of both parameters according to the Vollenweider and Kajak method (Vollenweider 1965; Kajak 2001). As indicated, the causes of water blooms in the area were municipal sources, i.e., waste (thrown away by tourists and residents) and agriculture (surface runoff of fertilizers and plant protection products from fields) (Buta et al. 2023; Smith et al. 1999).

## Conclusions

In the context of water restoration, the use of EM in separate parts of water reservoirs, as presented in the article, can be a more effective solution than their application on the entire surface of the reservoir. It results from increased eutrophication in shallow parts of such hydrographic objects. In this type of a separated ecosystem, the application of only EM, without other methods of renaturation, may prove to be long-term and effective in the context of the desired improvement of water quality parameters (both microbiological and physicochemical). Therefore, in the future, more comprehensive research should be conducted on this topic. Also, a larger number of similar research objects should be analyzed over a longer period of time. EM can be one of the effective methods to purify water from unfavorable microorganisms (HBN22, HBN36, CBN, FCBN, FEN). After the application of EM, a decrease in their concentration was found at an average level of 46.44 to 58.38%, consistent with other studies in this field. Statistical analysis showed the significance of the results for *p* ≤ 0.05. The following conclusions were drawn from the analyses described in this article:

1. Based on the tests performed, the average EM action duration ranged from 17.64 to 34.13 days, depending on the microbiological parameter considered.
2. After the application of EM, the average ecological status improved in terms of NO_3_–N, PO_4_–P, *TP*, and DO (by 6.12%, 31.03%, 7.92%, and 20.69%, respectively) and worsened in terms of pH and COD (by 23.78% and 7.06%, respectively). These results are statistically significant (*p* ≤ 0.05).
3. Comparing the series immediately before and after the application of EM, the percentage of samples meeting bathing water standards (defined by Polish regulations) increased by 400% concerning CBN. No differences were found for FEN.
4. The application of EM improved the trophic status of Turawa reservoir, expressed by the Carlson index, by 7.78%.
5. Principal component analysis showed that results were most similar for microbiological parameters (FEN, CBN, FCBN, HBN22, and HBN36), selected nutrients (*TP*, PO_4_–P, and *TN*), and some oxygen parameters (COD and BOD_5_).
6. According to the Spearman correlation matrix, a statistically significant, strong correlation (*R* ≥  ± 0.70 at *p* ≤ 0.05) occurred between the following parameters: HBN36/HBN22 (0.87), FCBN/CBN (0.80), NO_3_–N/EC (0.84), DO/EC (− 0.72), and DO/NO_3_–N (− 0.73).
7. The literature search showed that after applying EM, the CBN, FEN, NO_3_–N, PO_4_–P, and *TP* values decreased, and the pH increased. No consistent results were found for *TN*, EC, and DO.

There is a need for more research on the effectiveness of EM and its application in restoring eutrophic water reservoirs. The scope of research should include more physicochemical and microbiological parameters related to priority substances (e.g., heavy metals, specific organic pollutants) and biological parameters (e.g., ichthyofauna, plankton, benthos, macrophytes). In addition to water testing, it is also recommended to focus on testing bottom sediments which have strong sorbent properties for contaminants. Technically, it is possible to compare the effectiveness of EM applied at different times to different matrices (e.g., water, bottom sediments, plants) in different forms (e.g., as a liquid solution, loess balls, soil balls, mud balls, or Bokashi balls), at different concentrations and compositions, or with the use of other solutions to assist in water purification (physical, chemical, biological methods).

## Data Availability

The datasets used and/or analyzed during the current study are available from the corresponding author on reasonable request.
